# The PICK1 Ca^2+^ sensor modulates *N*-methyl-d-aspartate (NMDA) receptor-dependent microRNA-mediated translational repression in neurons

**DOI:** 10.1074/jbc.M117.776302

**Published:** 2017-04-12

**Authors:** Dipen Rajgor, Maria Fiuza, Gabrielle T. Parkinson, Jonathan G. Hanley

**Affiliations:** From the School of Biochemistry and the Centre for Synaptic Plasticity, University of Bristol, Bristol BS8 1TD, United Kingdom

**Keywords:** AMPA receptor (AMPAR), Argonaute, calcium, dendrite, synaptic plasticity

## Abstract

MicroRNAs (miRNAs) are important regulators of localized mRNA translation in neuronal dendrites. The presence of RNA-induced silencing complex proteins in these compartments and the dynamic miRNA expression changes that occur in response to neuronal stimulation highlight their importance in synaptic plasticity. Previously, we demonstrated a novel interaction between the major RNA-induced silencing complex component Argounaute-2 (Ago2) and the BAR (bin/amphiphysin/rvs) domain protein PICK1. PICK1 recruits Ago2 to recycling endosomes in dendrites, where it inhibits miRNA-mediated translational repression. Chemical induction of long-term depression via NMDA receptor activation causes the dissociation of Ago2 from PICK1 and a consequent increase in dendritic miRNA-mediated gene silencing. The mechanism that underlies the regulation of PICK1-Ago2 binding is unknown. In this study, we demonstrate that the PICK1-Ago2 interaction is directly sensitive to Ca^2+^ ions so that high [Ca^2+^]_free_ reduces PICK1 binding to Ago2. Mutating a stretch of C-terminal Ca^2+^-binding residues in PICK1 results in a complete block of NMDA-induced PICK1-Ago2 disassociation in cortical neurons. Furthermore, the same mutant also blocks NMDA-stimulated miRNA-mediated gene silencing. This study defines a novel mechanism whereby elevated [Ca^2+^] induced by NMDA receptor activation modulates Ago2 and miRNA activity via PICK1. Our work suggests a Ca^2+^-dependent process to regulate miRNA activity in neurons in response to the induction of long-term depression.

## Introduction

MicroRNAs (miRNAs)^2^ are small non-coding RNA species that regulate posttranscriptional gene silencing of specific mRNA targets (1). Their canonical mechanism of action involves imperfect base-pairing within the 3′ UTR of target mRNA transcripts, where they can be translationally repressed by the RNA-induced silencing complex (RISC) (2).

The brain expresses numerous different miRNAs that play important roles in development and the regulation of synaptic plasticity (3). Mice lacking Dicer or DGCR8, two proteins involved in miRNA biogenesis, display altered synaptic protein expression, synaptic transmission, dendritic spines, and defects in memory and learning (4, 5). Long-term potentiation (LTP) and long-term depression (LTD) are two forms of long-lasting plasticity that have been widely studied as mechanisms that underlie memory and learning (6, 7). LTP results in a persistent enhancement of synaptic transmission and enlargement of dendritic spines, whereas LTD is a contrasting process in which the efficacy of synaptic transmission is reduced and dendritic spines undergo shrinkage or complete elimination. Both forms of long-term plasticity are induced upon NMDA receptor (NMDAR) activation, which stimulates Ca^2+^ influx and trigger relevant signaling cascades (6).

In neurons, many miRNAs localize to dendrites and are regulated by synaptic activity. Some of these miRNAs have been shown to play regulatory roles in spine morphology by locally targeting actin-regulatory pathways and the expression of AMPA receptor (AMPAR) subunits (8–10). For instance, the chemical induction of LTD by NMDAR activation results in rapid and wide-ranging changes in the dendritic miRNAome that are required for long-lasting remodeling of dendritic spines (8). miR-191 expression is reduced in dendrites, resulting in up-regulation of tropomodulin-2 and stimulation of actin depolymerization, followed by shrinkage of dendritic spines (8). In contrast, expression of miR-501-3p increases in dendrites, where it represses GluA1 expression and is required for long-lasting remodeling of dendritic spines (9). As miRNAs play fundamental roles in synaptic plasticity, it is important to understand the molecular changes that occur within dendrites and spines that facilitate miRNA-mediated translational repression. Apart from changes in the expression level of specific miRNAs, the mechanisms for the transduction of NMDAR-mediated Ca^2+^ influx into changes in miRNA activity in neurons during synaptic plasticity are unknown.

PICK1 is a BAR (bin/amphiphysin/rvs) and PDZ domain protein that regulates the trafficking of AMPARs in LTD and LTP (11–13). Furthermore, PICK1 is a Ca^2+^-sensing protein that responds to NMDAR-mediated Ca^2+^ influx to regulate its interaction with the AMPAR subunit GluA2. The increased Ca^2+^ binding to PICK1 upon NMDAR activation results in an enhanced interaction between PICK1 and GluA2, which facilitates AMPAR internalization (14, 15). Previously, we reported a novel interaction between the core RISC regulator Ago2 and PICK1 on recycling endosomes in neuronal dendrites. We demonstrated a direct interaction between the C-terminal tail of PICK1 and the PIWI domain of Ago2. During NMDAR-mediated chemically induced LTD (cLTD), Ago2 is displaced from PICK1-containing recycling endosomes, resulting in enhanced miRNA activity of dendritically localized miRNAs (16). Because cLTD causes a reduction in Ago2-PICK1 complexes, we were interested in identifying the molecular mechanisms that underlie this response. As PICK1 binding to GluA2 is Ca^2+^-sensitive, we hypothesized that Ca^2+^ binding to PICK1 may also regulate its interaction with Ago2.

In this study, we demonstrate that the Ca^2+^-sensing property of PICK1 is required for regulating its interaction with Ago2. We show that, in high [Ca^2+^]_free_, PICK1-Ago2 complexes are disrupted. Furthermore, we show that, when the PICK1 Ca^2+^-binding site is mutated, the cLTD-induced increase in the activity of dendritically localized miRNAs in neurons is blocked. This study describes a novel mechanism for the regulation of Ago2 activity by Ca^2+^ in response to NMDAR activation in neurons, which we propose might be involved in initiating changes in protein expression required for LTD.

## Results

### PICK1-Ago2 interaction is regulated by Ca^2+^

**Figure 1.**
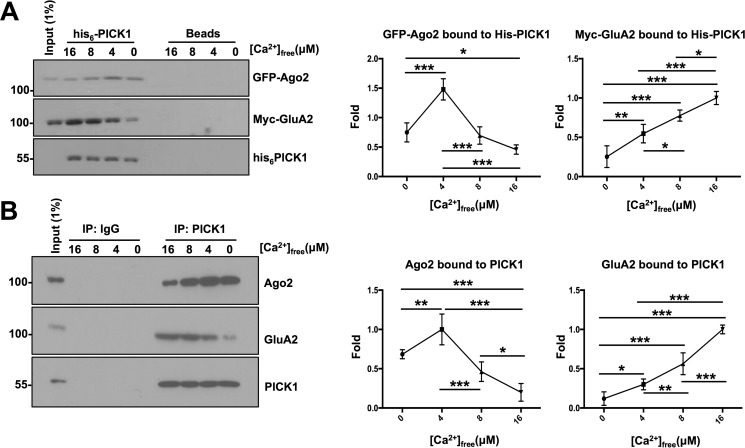
**PICK1 interacts with Ago2 and GluA2 in a Ca^2+^-dependent manner.** *A*, recombinant His_6_-PICK1 pulldowns performed from HEK293 cells co-transfected with GFP-Ago2 and Myc-GluA2 under defined [Ca^2+^]_free_. Bound proteins were detected by Western blotting. His_6_-PICK1 had the strongest binding with GFP-Ago2 at 4 μm [Ca^2+^]_free_ and with Myc-GluA2 at 16 μm [Ca^2+^]_free_. *n* = 5; *, *p* < 0.05; **, *p* < 0.01; ***, *p* < 0.001; one-way ANOVA, Dunnett's post hoc test. *B*, endogenous PICK1 was immunoprecipitated from DIV14 cortical neuron lysate in defined [Ca^2+^]_free_. Reduced Ago2 and increased GluA2 interactions were observed with PICK1 under high [Ca^2+^]_free_. *n* = 5; *, *p* < 0.05; **, *p* < 0.01; ***, *p* < 0.001; one-way ANOVA, Dunnett's post hoc test.

PICK1 has been identified previously as a Ca^2+^ sensor that interacts with the AMPAR subunit GluA2 in a Ca^2+^-dependent manner (14). Because the PICK1-Ago2 interaction is reduced upon NMDAR-mediated cLTD (16), we investigated whether Ca^2+^ modulates PICK1-Ago2 binding. First, we incubated His_6_-PICK1 immobilized on nickel beads with extract from HEK293 cells co-expressing GFP-Ago2 and Myc-GluA2 in buffers containing defined [Ca^2+^]_free_ of 0, 4, 8, and 16 μm (Fig. 1*A*). We chose these [Ca^2+^]_free_ because PICK1 shows Ca^2+^ regulated binding properties over this range of [Ca^2+^]_free_ for GluA2 (14). Indeed, His_6_-PICK1 binding to Myc-GluA2 significantly increased with increasing [Ca^2+^]_free_, as demonstrated previously (14). In contrast, PICK1 binding to GFP-Ago2 initially increased as the [Ca^2+^]_free_ was increased from 0–4 μm but then became significantly weaker as the [Ca^2+^]_free_ further increased to 16 μm (Fig. 1*A*).

Next we examined whether endogenous PICK1-Ago2 protein complexes are regulated by the availability of [Ca^2+^]_free_. PICK1 immune complexes from DIV14 cortical neuronal extracts were isolated under varying [Ca^2+^]_free_ (Fig. 1*B*). As with our His_6_ pulldown experiments, we observed a significant increase in Ago2 binding to PICK1 when the [Ca^2+^]_free_ was increased from 0 to 4 μm but a significant decrease at 16 μm [Ca^2+^]_free_. Consistent with previous findings, more GluA2 bound to PICK1 isolated under higher [Ca^2+^]_free_ (14).

**Figure 2.**
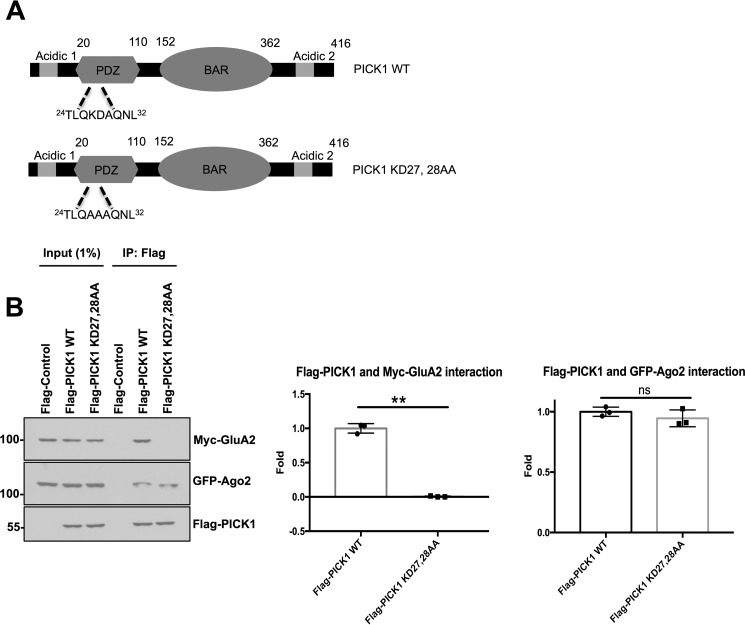
**Ago2 and GluA2 binding to PICK1 is not mutually exclusive.** *A*, schematic illustrating the location of amino acids Lys-27 and Asp-28 mutated to Ala in the PICK1 K27A,D28A mutant. *B*, FLAG-PICK1 K27A,D28A did not bind Myc-GluA2 but bound GFP-Ago2 at a similar level as FLAG-PICK1 WT. FLAG control cells were transfected with an empty FLAG vector, Myc-GluA2, and GFP-Ago2. Co-IPs were performed in IP buffer. Bound proteins were detected by Western blotting. The graph shows quantification of Myc-GluA2 and GFP-Ago2 binding to FLAG-PICK1 WT and FLAG-PICK1 K27A,D28A. *n* = 3; *ns*, not significant; **, *p* < 0.01, one-way ANOVA, Dunnett's post hoc test.

Because Ago2 and GluA2 bind PICK1 with approximately opposing [Ca^2+^] dependence, we investigated whether Ago2 and GluA2 bind to PICK1 in a mutually exclusive manner. We used PICK1 carrying two point mutations (K27A,D28A) in the PDZ domain that disrupt GluA2 binding (Fig. 2*A*) (17). In co-IPs from HEK293 cells triple-transfected with GFP-Ago2, Myc-GluA2, and FLAG-PICK1 WT or FLAG-PICK1 K27A,D28A, the PICK1-GluA2 interaction was completely abolished by the K27A,D28A mutation, as shown previously (17, 18). In contrast, the Ago2 interaction with PICK1 K27A,D28A was indistinguishable from its interaction with PICK1 WT, demonstrating that the interaction between Ago2 and PICK1 is not affected by GluA2 binding to PICK1 (Fig. 2*B*).

### The PICK1 C-terminal acidic region regulates Ago2 binding

**Figure 3.**
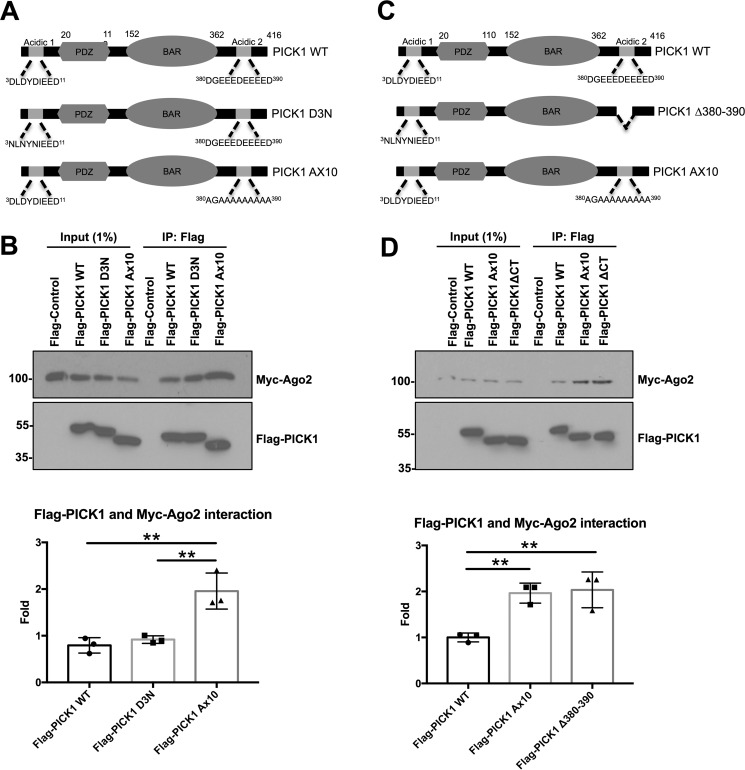
**The C-terminal acidic region of PICK1 regulates Ago2 interaction.** *A*, schematic illustrating the location of the acidic regions in PICK1 that bind Ca^2+^. *B*, Myc-Ago2 bound significantly more FLAG-PICK1 A×10 compared with WT or the D3N mutant from transfected HEK293 cell extracts. FLAG control cells were co-transfected with an empty FLAG vector and Myc-Ago2. Co-IPs were performed in IP buffer. Bound proteins were detected by Western blotting. *n* = 3; **, *p* < 0.01, one-way ANOVA, Dunnett's post hoc test. *C*, schematic illustrating the deletion of residues 380–390 in PICK1 Δ380–390. *D*, FLAG-PICK1 A×10 and Δ380–390 bound similar levels of Myc-Ago2 from transfected HEK293 cell extracts. FLAG-control cells were co-transfected with an empty FLAG vector and Myc-Ago2. Co-IPs were performed in IP buffer. *n* = 3; **, *p* < 0.01, one-way ANOVA, Dunnett's post hoc test.

PICK1 contains two acidic regions that have been demonstrated previously to bind Ca^2+^ (14, 15). One is located at the N terminus between amino acids 3–11, and the second site is positioned between residues 380–390, toward the C terminus of PICK1 and within the region of PICK1 required for Ago2 binding (16). To identify which site was required for regulating the Ca^2+^-sensitive interaction with Ago2, we examined the association of Ago2 with PICK1 Ca^2+^-insensitive mutants. It has been shown previously that mutating the first three acidic amino acids in the N-terminal acidic stretch to asparagine (D3N) renders the N-terminal site insensitive to Ca^2+^ (15). The specific residues required for Ca^2+^ binding between 380–390 have not yet been determined. Therefore, we mutated all 10 acidic residues to alanine (A×10) to prevent the C-terminal region from binding Ca^2+^ (Fig. 3*A*).

When FLAG-tagged versions of these mutants were co-transfected into HEK293 cells with Myc-Ago2, co-immunoprecipitations showed that FLAG-PICK1 D3N bound Myc-Ago2 at similar levels compared with FLAG-PICK1 WT. Interestingly, the interaction of Ago2 with FLAG-PICK1 A×10 was significantly greater compared with FLAG-PICK1 WT and D3N (Fig. 3*B*), suggesting that the C-terminal acidic region of PICK1 regulates Ago2 binding. To confirm that the C-terminal acidic region of PICK1 regulates Ago2 binding, we performed co-IP experiments in HEK293 cells co-transfected with Myc-Ago2 and FLAG-PICK1 Δ380–390, in which residues 380–390 were deleted (Fig. 3*C*). FLAG-PICK1 Δ380–390 showed a similar interaction with Myc-Ago2 as FLAG-PICK1 A×10 (Fig. 3*D*).

**Figure 4.**
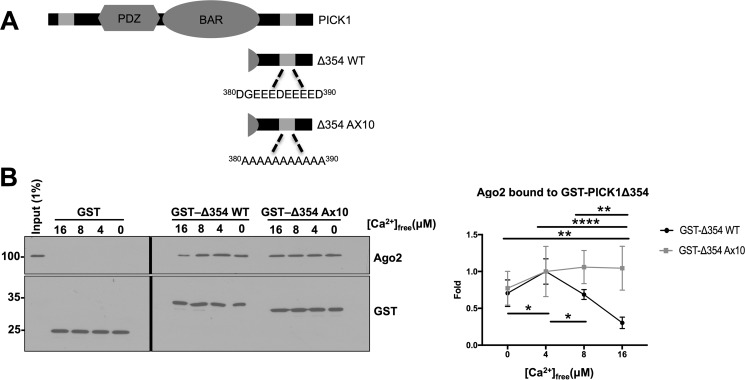
**GST-PICK1 Δ354 binds endogenous Ago2 from cortical neuronal extract in a Ca^2+^-dependent manner.** *A*, schematic illustrating PICK1 Δ354. *B*, GST-Δ354 WT and A×10 pulldowns performed from DIV14 cortical neuronal lysates in different [Ca^2+^]_free_. Bound proteins were detected by Western blotting. *n* = 4; **, *p* < 0.01; ****, *p* < 0.0001; two-way ANOVA, Bonferroni post hoc test.

To investigate the role of PICK1 residues 380–390 in Ca^2+^-dependent Ago2 binding, we performed GST pulldown experiments on cortical neuronal extracts in defined [Ca^2+^]_free_ using the C-terminal tail of PICK1 (GST-Δ354) as bait (Fig. 4*A*). Ago2 binding to GST-Δ354 displayed a biphasic dependence on [Ca^2+^]_free_, consistent with our pulldown and co-IP experiments shown in Fig. 1. The strongest interaction was observed at 4 μm [Ca^2+^]_free_ and the weakest at 16 μm [Ca^2+^]_free_. Interestingly, Ago2 binding to GST-Δ354 A×10 did not respond to changes in [Ca^2+^]_free_, indicating that the Ca^2+−^sensitive Ago2-PICK1 interaction requires amino acids 380–390 of PICK1 (Fig. 4*B*). Together, these experiments demonstrate that the PICK1-Ago2 interaction is regulated by Ca^2+^ binding to the C-terminal acidic region of PICK1 located within the Ago2 binding domain.

### The PICK1 C-terminal acidic region mediates Ago2 dissociation in response to NMDAR-mediated cLTD

Previously, we demonstrated that the PICK1-Ago2 interaction is significantly reduced after the induction of cLTD (16). During cLTD, Ca^2+^ entry through NMDARs increases localized intracellular [Ca^2+^]. Therefore, to determine whether the Ca^2+^-sensing property of PICK1 is required for mediating the disassociation of Ago2 during cLTD, we performed molecular replacement experiments in cortical neurons, where endogenous PICK1 was knocked down with a previously characterized shRNA for PICK1 and replaced with sh-resistant GFP-PICK1 WT or GFP-PICK1 A×10 (15, 16). shPICK1 robustly reduced the expression of endogenous PICK1 by ∼80% in neuronal dendrites, whereas shRNA-resistant GFP-PICK1 WT and GFP-PICK1 A×10 restored PICK1 expression to endogenous levels (supplemental Fig. S1). To investigate whether these constructs altered the neuronal architecture, we performed a Sholl analysis to measure dendritic branching. PICK1 knockdown decreased the number of dendritic intersections 70–100 μm away from the cell body in a similar manner to a previous report (19). This phenotype was restored to control levels by either GFP-PICK1 WT or A×10 (supplemental Fig. S2, *A* and *B*). Furthermore, neither PICK1 knockdown nor molecular replacement altered total dendritic length (supplemental Fig. S2*C*). Together, these experiments suggest that the C-terminal Ca^2+^-binding properties of PICK1 do not regulate dendritic branching or dendritic length.

**Figure 5.**
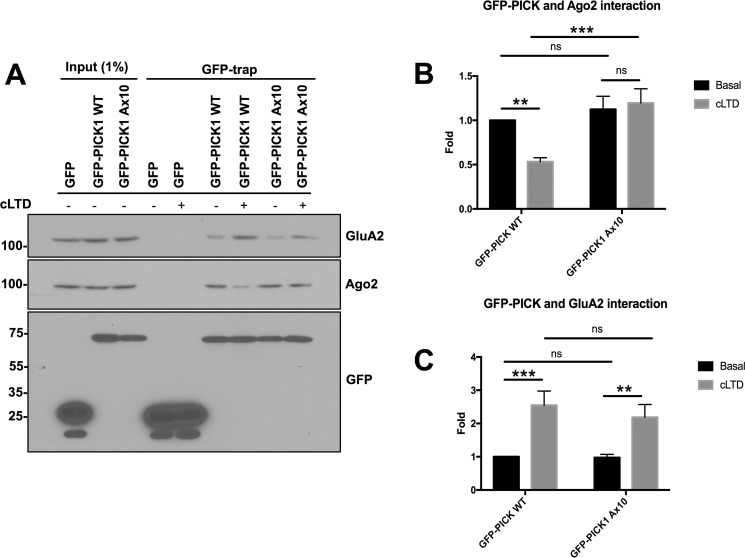
**The PICK1 A×10 mutation blocks the cLTD-induced decrease in the Ago2-PICK1 interaction in neurons.** *A*, GFP-trap was used to capture GFP-PICK1 WT and GFP-PICK1 A×10 complexes before and after NMDAR-induced cLTD, and bound proteins were detected by Western blotting. *B*, cLTD induction caused a significant reduction in Ago2 binding to GFP-PICK WT, which was blocked by the A×10 mutation. *C*, cLTD induction caused a significant increase in GluA2 binding to both GFP-PICK1 WT and GFP-PICK1 A×10. *n* = 3; *ns*, not significant; **, *p* < 0.01; ***, *p* < 0.001; two-way ANOVA, Bonferroni post hoc test.

We performed GFP-trap experiments to measure the interaction between GFP-PICK1 and endogenous Ago2 under basal and cLTD conditions. Under basal conditions, similar levels of Ago2 bound to PICK1 WT and A×10 (Fig. 5, *A* and *B*). 10 min after cLTD induction, Ago2 binding to PICK1 WT was significantly reduced, as seen previously (16). However, the interaction between PICK1 A×10 and Ago2 was not disrupted by cLTD (Fig. 5, *A* and *B*). In contrast, cLTD caused a similar increase in GluA2 interaction with both PICK1 WT and the A×10 mutant (Fig. 5, *A* and *C*). These data indicate that the Ca^2+^-sensing property of the PICK1 C-terminal acidic region underlies the release of Ago2 from PICK1 following cLTD induction but that it is not involved in regulating the interaction with AMPARs.

**Figure 6.**
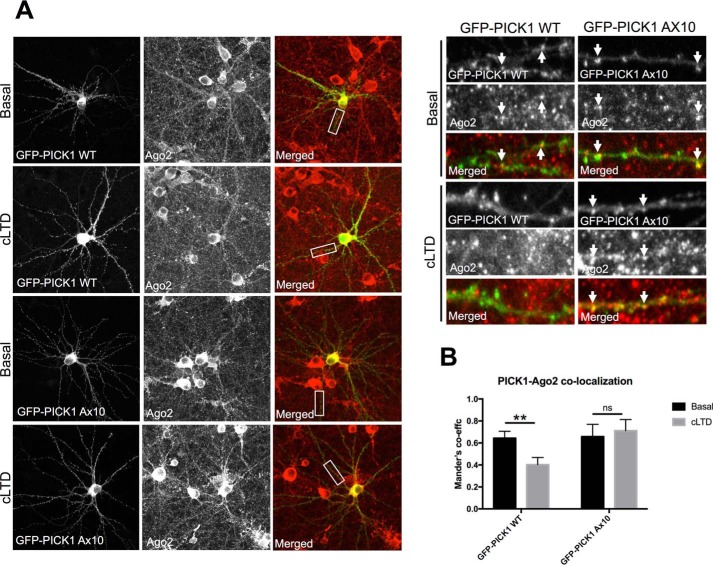
**The PICK1 A×10 mutation blocks the cLTD-induced decrease in Ago2-PICK1 co-localization in neuronal dendrites.** *A*, cLTD induction caused a reduction in co-localization between endogenous Ago2 and GFP-PICK1 WT, which was blocked by the A×10 mutation. *Arrows* indicate overlapping foci. *B*, quantification of co-localization of GFP-PICK1 and Ago2 was determined by Mander's coefficients. *n* = 5; *ns*, not significant; **, *p* < 0.01, two-way ANOVA, Bonferroni post hoc test.

PICK1 localizes to endosomal compartments (20), and we showed previously that Ago2 is present with PICK1 on recycling endosomes and that Ago2 dissociates from PICK1-containing compartments in response to cLTD induction (16). We therefore hypothesized that Ca^2+^ sensing by PICK1 triggers Ago2 release from PICK1-positive endosomes. To test this, we examined Ago2 co-localization with GFP-PICK1 WT or A×10 under basal or cLTD conditions. Although we did not detect any difference in the degree of co-localization of Ago2 with GFP-PICK1 WT or A×10 under basal conditions, cLTD caused a significant reduction in co-localization between GFP-PICK1 WT and Ago2, which was completely blocked by the A×10 mutation (Fig. 6, *A* and *B*). Together, these experiments demonstrate that the C-terminal acidic region of PICK1 is essential for regulating the disassociation of PICK1 from Ago2 in response to cLTD.

**Figure 7.**
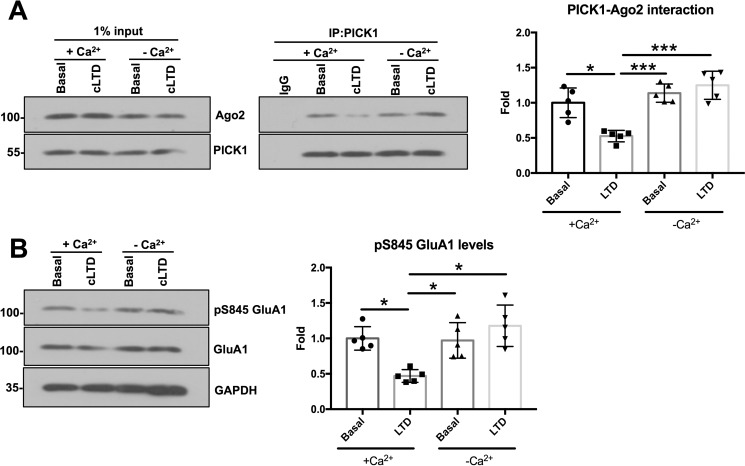
**Extracellular Ca^2+^ is required for cLTD-induced dissociation of Ago2 from PICK1 in cultured cortical neurons.** *A*, cLTD induction caused Ago2-PICK1 dissociation 10 min after stimulation only in the presence of extracellular Ca^2+^ (1.8 mm). Endogenous PICK1 was immunoprecipitated from DIV14 cortical neuron lysate in IP buffer, and bound proteins were detected by Western blotting. *n* = 5; *, *p* < 0.05; ***, *p* < 0.0001; one-way ANOVA, Dunnett's post hoc test. *B*, dephosphorylation of serine 845 of GluA1 in a Ca^2+^-dependent manner confirms successful cLTD induction. Phosphorylated GluA1 was quantified by normalizing to total GluA1. *n* = 5; *, *p* < 0.05, one-way ANOVA, Dunnett's post hoc test.

To investigate whether the dissociation of Ago2 from PICK1 caused by cLTD induction required extracellular Ca^2+^, we examined endogenous PICK1-Ago2 binding in cortical neurons stimulated with NMDA in the absence of extracellular Ca^2+^. NMDAR activation significantly reduced PICK1-Ago2 binding by ∼50% in the presence of normal (1.8 mm) Ca^2+^. In contrast, NMDAR stimulation in the absence of extracellular Ca^2+^ had no effect on PICK1-Ago2 binding, indicating that extracellular Ca^2+^ is the primary source of Ca^2+^ that disrupts PICK1-Ago2 interactions during cLTD (Fig. 7*A*). We also confirmed that our stimulation protocol induced cLTD by analyzing GluA1 phosphorylation at serine 845. Dephosphorylation at this site by the Ca^2+^-dependent phosphatase calcineurin is a hallmark of LTD (21, 22). GluA1 phosphorylation at serine 845 decreased in response to NMDAR stimulation in the presence of extracellular Ca^2+^ but was unchanged in the absence of extracellular Ca^2+^ (Fig. 7*B*). This demonstrated successful induction of cLTD in the presence of extracellular Ca^2+^.

**Figure 8.**
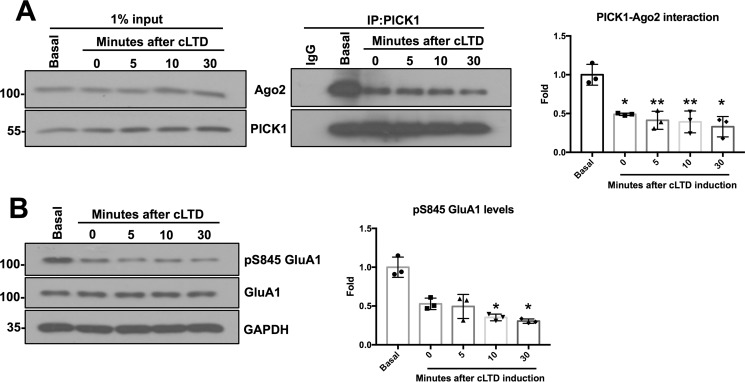
**Ago2-PICK1 complexes dissociate immediately after cLTD.** *A*, endogenous PICK1 was immunoprecipitated from DIV14 cortical neuron lysate in IP buffer, and bound proteins were detected by Western blotting. Lysates were prepared either without NMDA treatment (basal) or 0, 5, 10, 30 min after 3-min NMDA treatment. The graph shows quantification of Ago2-PICK interaction before and after cLTD. *n* = 3; *, *p* < 0.05; **, *p* < 0.01; one-way ANOVA, Dunnett's post hoc test. *B*, dephosphorylation of serine 845 of GluA1 in a time-dependent manner confirms successful cLTD induction. Phosphorylated GluA1 was quantified by normalizing to total GluA1. *n* = 3; *, *p* < 0.05, one-way ANOVA, Dunnett's post hoc test.

To further define the dissociation of Ago2 from PICK1 in response to cLTD induction, we performed a time course experiment to analyze endogenous PICK1-Ago2 complexes at 0, 5, 10, and 30 min after NMDAR stimulation. Interestingly, a significant reduction in PICK1-Ago2 binding was observed immediately after stimulation with no further reduction being observed at the later time points (Fig. 8*A*). Serine 845 dephosphorylation followed a similar time course (Fig. 8*B*). Together, these experiments suggest that extracellular Ca^2+^ entry through NMDARs disrupts PICK1-Ago2 complexes very rapidly after stimulation.

### PICK1 Ca^2+^ sensing is required for miRNA-mediated gene silencing caused by cLTD

**Figure 9.**
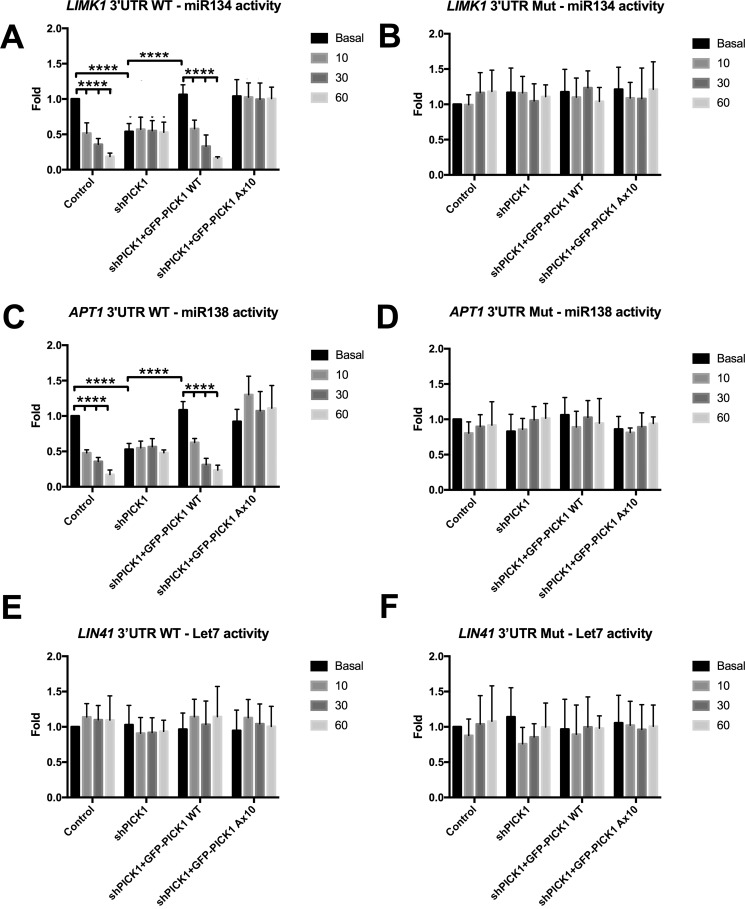
**The PICK1 A×10 mutation blocks the cLTD-induced increase in gene silencing.** *A*, miR134-*Limk1* 3′ UTR. A Dual-Luciferase assay was performed in neurons expressing *Renilla* and firefly luciferase reporter containing the *Limk1* 3′ UTR. Replacement of endogenous PICK1 with GFP-PICK1 WT or GFP-PICK1 A×10 has no effect on miR134 activity under basal conditions; however, GFP-PICK1 Ax10 blocks the cLTD-induced increase in miR134 activity at 10, 30, and 60 min after stimulation. *B*, manipulations have no effect on luciferase reporter expression when the miR134-binding site on the LIMK1 3′ UTR is abolished by mutation. *C*, miR138-*APT1* 3′ UTR. A Dual-Luciferase assay was performed in neurons expressing *Renilla* and firefly luciferase reporter containing *APT1* 3′ UTR. Replacement of endogenous PICK1 with GFP-PICK1 WT or GFP-PICK1 A×10 has no effect on miR138 activity under basal conditions; however, GFP-PICK1 A×10 blocks the cLTD-induced increase in miR138 activity at 10, 30, and 60 min after stimulation in a similar manner as shown in *A. D*, manipulations have no effect on luciferase reporter expression when the miR138 binding site on the APT1 3′ UTR is abolished by mutation. *E* and *F*, Let7-*LIN41* 3′ UTR. A Dual-Luciferase assay was performed in neurons expressing *Renilla* and firefly luciferase reporter containing the *LIN41* 3′ UTR. Let7 activity is unaffected by cLTD induction or manipulation of PICK1. *n* = 3; ****, *p* < 0.0001, two-way ANOVA, Bonferroni post hoc test.

We reported previously that cLTD enhances miRNA-mediated gene silencing of the dendritically localized miR134 in a PICK1-dependent manner (16), suggesting that PICK1 is an inhibitor of Ago2 function under basal conditions. To investigate whether the C-terminal Ca^2+^ binding region of PICK1 is involved in the cLTD-induced increase in Ago2 function, we measured the activity of a number of miRNAs using luciferase reporter assays. The 3′ UTRs of *LIMK1*, *APT1*, and *LIN41* contain miRNA binding sites for miR134, miR138, and Let7, respectively (23–25). When these UTRs are fused to DNA encoding luciferase, its expression can be regulated in a miRNA-dependent manner. MiR134 and miR138 are expressed primarily in neuronal dendrites, whereas Let7 is ubiquitously expressed throughout neurons (23–26). PICK1 knockdown reduced luciferase expression from the *LIMK1* reporter under basal conditions, which was rescued by co-expression of GFP-PICK1 WT and also by GFP-PICK1 A×10 (Fig. 9*A*), indicating that the A×10 mutation has no effect on miRNA activity under basal conditions. In response to cLTD induction, we observed a ∼50% reduction in luciferase activity 10 min after stimulation, as reported previously (16). Gene silencing increased further at later time points, with ∼70% reduction in luciferase activity at 30 min and ∼90% reduction at 60 min after NMDAR stimulation in both control and GFP-PICK1 WT-expressing cells. The cLTD-induced increase in gene silencing was completely blocked by expression of GFP-PICK1 A×10. The same findings were observed with the *APT1* 3′ UTR luciferase reporter, indicating that miR138 activity is regulated by the C-terminal PICK1 Ca^2+^-binding region in a similar manner (Fig. 9*C*). Luciferase activity was not significantly affected under basal or cLTD conditions with any PICK1 knockdown or replacement background when the miR134- and miR138-binding sites were mutated in the *LIMK1* and *APT1* reporter constructs so that they could no longer bind miRNA (Fig. 9, *B* and *D*). PICK1 knockdown or molecular replacement with GFP-PICK1 A10 did not alter Let7 activity, as measured using the *LIN41* luciferase reporter under basal or cLTD conditions (Fig. 9, *E* and *F*). Taken together, these experiments suggest that the C-terminal Ca^2+^-binding region of PICK1 regulates dendritic miRNA-mediated gene silencing events in response to cLTD induction.

## Discussion

Here we have identified a novel molecular mechanism that regulates the interaction between PICK1 and the miRISC protein Ago2. We showed previously that PICK1 inhibits Ago2 function in neuronal dendrites and that, during cLTD, Ago2 is released from PICK1, causing an increase in miRNA-mediated translational repression (16). In this study, we show that the PICK1-Ago2 interaction is regulated directly by the ability of PICK1 to bind Ca^2+^ ions. We demonstrate that amino acids 380–390 in PICK1 are responsible for regulating the Ago2-PICK1 interaction in a Ca^2+^ dependent manner and that mutating this C-terminal Ca^2+^-binding region of PICK1 blocks miR134- and miR138-dependent translational repression in response to cLTD induction.

### Ca^2+^ sensitivity of the PICK1-Ago2 interaction

Our results show that the PICK1-Ago2 interaction is significantly reduced at high [Ca^2+^]_free_ of 16 μm compared with zero Ca^2+^. However, the interaction is strongest at 4 μm [Ca^2+^]_free_, suggesting a biphasic dependence on [Ca^2+^]. The PICK1-GluA2 interaction also has a biphasic dependence on [Ca^2+^], but, in contrast to PICK1-Ago2, binding increases between 0 and 15 μm, with decreased PICK1-GluA2 interaction again at 30 μm [Ca^2+^]_free_ (14). This difference is likely to reflect the distinct Ca^2+^-binding sites involved in regulating GluA2 and Ago2 interactions and suggests that PICK1 mediates a variety of complex biochemical responses to changes in [Ca^2+^]_free_. The intracellular [Ca^2+^] reached during LTD induction is unclear. Our results show that PICK1 binding to Ago2 decreased and binding to GluA2 increased during cLTD induction in neurons. In binding experiments using purified components, we could mimic these effects at 16 μm [Ca^2+^]_free_, suggesting that the biochemical changes observed *in vitro* at 16 μm [Ca^2+^]_free_ may occur in neurons during NMDAR-mediated cLTD. However, it is important to note that Ca^2+^ buffering in our *in vitro* setting presumably does not mimic Ca^2+^ buffering in dendritic spines.

PICK1 contains two acidic regions that are capable of binding Ca^2+^ ions (14). We show that, although the N-terminal acidic region is not involved in Ago2 regulation, mutating the C-terminal acidic region of PICK1 (380–390) blocks dissociation from Ago2. A possible explanation for the effect of Ca^2+^ is that Ca^2+^ neutralizes the negative charge of the PICK1 acidic region. This predicts that the neutral A×10 mutant would mimic the Ca^2+^-bound state and have reduced affinity for PICK1, as observed for PICK1 WT, at high [Ca^2+^]_free_. However, we suggest that, at 16 μm [Ca^2+^]_free_, the acidic motif still retains some negative charge, which may explain why Ago2-PICK1 WT binding at 16 μm does not coincide with the PICK1 A×10-Ago2 at zero [Ca^2+^]. Moreover, Ago2 binding to A×10 PICK1 was similar to PICK1 WT in neurons under basal conditions, and, in heterologous cells, we observed an enhanced association of A×10 PICK1 with Ago2 compared with PICK1 WT. We speculate that this difference between heterologous cells and neurons under basal conditions is due to neurons expressing numerous PICK1 interactors such as GluA2 and other neuron-specific channels, transporters, etc. (27, 28) that are not expressed in HEK293 cells. These proteins may influence the manner in which Ca^2+^ regulates PICK1 binding to Ago2. Hence we propose that the optimal [Ca^2+^] for promoting Ago2-PICK1 binding is slightly different in neurons compared with the heterologous system.

### Ca^2+^-sensitive PICK1-Ago2 binding in synaptic plasticity

We demonstrate a Ca^2+^-sensitive mechanism for regulating miRNA function in neurons. In response to cLTD, the activity of dendritically enriched miR134 is enhanced in neuronal dendrites and spines to promote silencing of *LIMK1* (16, 23). PICK1 A×10 blocks the cLTD-induced increase in miR134 activity, suggesting that Ca^2+^ releases the inhibitory effects of PICK1 on Ago2 during cLTD.

**Figure 10.**
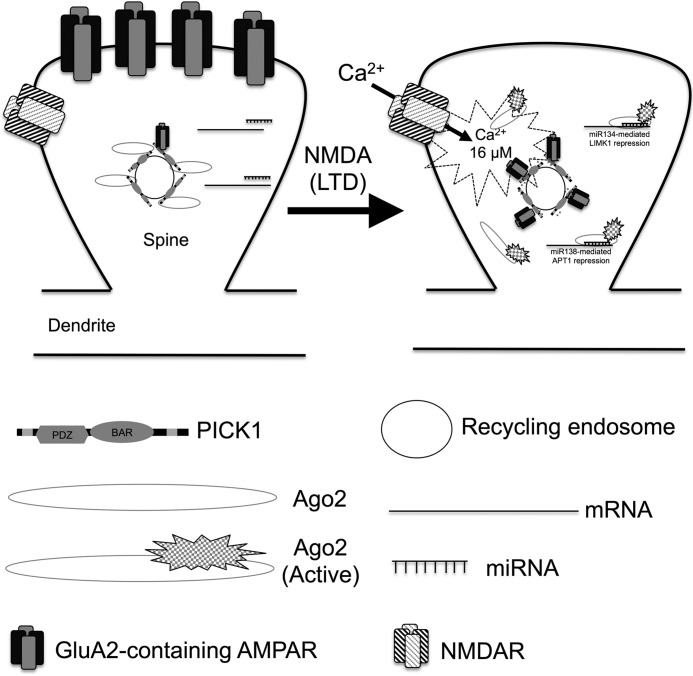
**PICK1 is a Ca^2+^ sensor for GluA2-containing AMPARs and Ago2.** PICK1 contains two Ca^2+^-binding regions that regulate interactions with GluA2 and Ago2. Under basal conditions, when [Ca^2+^] is low in dendritic spines, Ago2 is recruited to recycling endosomes by the C-terminal domain of PICK1. Upon cLTD induction, localized intracellular [Ca^2+^] increases, and Ca^2+^ binding to PICK1 releases Ago2 and increases its interaction with GluA2-containing AMPARs to promote their internalization. The released Ago2 causes increased miRISC activity of miR134 on LIMK1 and miR138 on APT1.

During NMDAR-mediated cLTD, a Ca^2+^ gradient is likely to exist within dendritic spines, where the [Ca^2+^]_free_ is highest toward the surface of spines in microdomains close to Ca^2+^ channels (29). This is where PICK1 is likely to have the strongest binding properties for GluA2 to facilitate its internalization (14). Recycling endosomes closely associated with dendritic spines close to synapses (30, 31) might also be where Ago2 is released by PICK1 (Fig. 10). Furthermore, NMDAR activation at the cell surface has been demonstrated to provoke the release of Ca^2+^ from Ryanodine receptor-associated Ca^2+^ stores such as the endoplasmic reticulum, which are present within dendrites and spines (32, 33) and are likely to contribute to increases in cytosolic [Ca^2+^] to attenuate PICK1-Ago2 interactions and, consequently, elevate Ago2 activity. This type of Ca^2+^ gradient-controlled miRNA-mediated gene silencing could allow neurons to control localized Ago2 activation upon stimulation and provide neurons with a unique mechanism to control localized protein synthesis. In this study, we were able to show that the original source of Ca^2+^ was extracellular because removal of Ca^2+^ during cLTD induction blocked the dissociation of PICK1 from Ago2.

Our results show that PICK1-mediated Ca^2+^ signaling during cLTD is responsible for increasing miRNA-mediated gene silencing of miR134 and miR138 but not Let7. This suggests the Ago2 bound to PICK1 only regulates a subset of miRNA-mediated translational repression events within neurons. miR134 and miR138 are enriched within dendrites, whereas Let7 is ubiquitously expressed throughout neurons (23–26). We propose that PICK1 specifically regulates the activity of dendritically localized miRNAs to mediate changes in protein expression required for synaptic plasticity. Measuring the activity of a wider range of miRNAs that are expressed in dendrites and/or the cell body will provide a better understanding of the role of PICK1 in miRNA-mediated gene silencing.

PICK1 knockdown mimics and occludes the cLTD-induced increase in miRNA activity at 10 min after stimulation, which is consistent with our observation that cLTD causes a very rapid dissociation of PICK1 from Ago2. However, our results show that cLTD causes a further increase in miRNA activity at later time points, indicating that additional mechanisms independent of PICK1 are involved in increasing miRNA activity in response to cLTD induction. It is interesting to note that, although we observed an ∼50% reduction in luciferase activity within 10 min of NMDAR activation, it has been suggested previously that the half-life of luciferase in HEK293 cells is ∼1–2 h (34). Our results indicate that luciferase has a shorter half-life (in the order of 10 min) in neurons, highlighting luciferase assays as an ideal tool for measuring rapid, activity-dependent changes in expression.

Whether the modulation of miRNA activity by PICK1 is required for LTD expression remains an unanswered question. AMPAR endocytosis occurs a few minutes after NMDAR stimulation (35), too soon to be affected by miRNA activity. Later events in LTD expression include dendritic spine shrinkage, which may involve down-regulation of LIMK1 (36). AMPAR internalization in response to LTD induction depends on an NMDAR-dependent increase in PICK1-GluA2 binding (37), and our results show that this is unaffected by the A×10 mutation. Therefore, in neurons expressing A×10 PICK1, the direct effect of PICK1 on NMDAR-dependent AMPAR trafficking should be the same as in control or WT PICK1-expressing neurons. Further experiments are necessary to determine which aspects of LTD are affected by the PICK1-dependent regulation of miRNA activity.

In conclusion, we have identified a novel mechanism for the regulation of miRNA activity by PICK1 in neurons. To our knowledge, this is the first mechanism to explain how an increase in [Ca^2+^]_free_ following NMDAR activation during the induction of synaptic plasticity causes an increase in RISC activity. Future work will determine the role of this mechanism in various kinds of learning and memory processes and, possibly, also in neurological diseases that involve changes in synaptic function.

## Experimental procedures

### DNA constructs

FLAG-PICK1 D3N, A×10, and K27A,D28A were created using site-directed mutagenesis of FLAG-PICK1 WT. FLAG-PICK1Δ380–390, Myc-Ago2, and GFP-Ago2 were reported previously (14, 16). Myc-GluA2 was kindly provided by Prof. J. Henley. Myc- and FLAG-tagged proteins were expressed in HEK293 cells from pcDNA3.1. GFP-PICK1 molecular replacement constructs were created by cloning shPICK1-resistant PICK1 WT and the A×10 mutant along with shPICK1 into pXLG3-GFP (a kind gift from Prof. J. Henley). The PICK1 ORF was fused to the C terminus of GFP and driven by the spleen focus-forming virus (SFFV) promoter. The H1 promoter and shPICK1 sequence were PCR-amplified from FUGW shPICK (16) and cloned into the KpnI site of pXLG3-GFP. shPICK1 has been characterized previously (15, 16). Luciferase-*LIMK1*, *APT1*, and *LIN41* 3′ UTRs were kindly provided by Prof. G. Schratt.

### HEK293 cell and cortical neuronal cultures

HEK293 cells were cultured in complete DMEM (Gibco) and passaged at ∼70–80% confluency. HEK cells were transfected with Jetpei (PolyPlus) transfection reagent according to the instructions of the manufacturer. Rat embryonic cortical neuronal cultures were prepared from embryonic day 18 Wistar rats using standard procedures. The culture medium was Neurobasal medium (Gibco) supplemented with B27 (Gibco) and 2 mm Glutamax. Neurons were plated at densities of 100, 000 cells/well of a 24-well plate, 500,000 cells/well of a 6-well plate, and 900,000 cells/6-cm dish. Neurons were transfected with plasmid DNA at days *in vitro* (DIV) 10–13 (unless otherwise stated) using Lipofectamine 2000 (Invitrogen) and used for experiments 2–5 days post-transfection. Chemical LTD was induced by bath application of 50 μm NMDA plus 20 μm glycine in HBS (140 mm NaCl, 5 mm KCl, 25 mm Hepes, 1.8 mm CaCl_2_, 0.8 mm MgCl_2_, and 10 mm glucose (pH 7.4)) for 3 min at 37 °C and harvested or fixed 10, 30, or 60 min later for biochemistry, luciferase assays, or imaging. Where cLTD was induced in the absence of Ca^2+^, cortical cultures were washed twice with HBS containing zero CaCl_2_ before bath application of 50 μm NMDA in the same buffer.

### Confocal microscopy and image analysis

Cells grown on coverslips were fixed in 4% paraformaldehyde (Thermo Fisher) in PBS (Sigma) supplemented with 2% sucrose at room temperature. Cells were permeabilized in 0.5% Nonidet P-40 for 2 min. Coverslips were blocked in 3% BSA (Sigma) for 1 h and incubated with anti-PICK1 (Abcam, ab3420; dilution, 1:100) in 3% BSA for 1 h at room temperature, followed by goat anti-rabbit Alexa Fluor 568 for 45 min (Thermo Fisher, A-11011; dilution, 1:1000) and mounted in DAPI-free medium (Sigma) onto slides. Coverslips were imaged on a Leica SP5 confocal system under a ×63/1.4 numerical aperture (NA) oil immersion or ×40/1.25 numerical aperture (NA) oil immersion objective using filters set up to image GFP, mRUBY, or Alexa Fluor 568. The Leica application suite software was used to acquire 0.37-μm stepped Z stacks throughout the depth of the cells, and maximal intensity projections generated by ImageJ Fiji are presented in this work. Image processing and co-localization analyses were performed using ImageJ and Coloc2. Three randomly selected dendrites were analyzed per neuron. Mander's fractions were measured using thresholding, values were normalized for each of at least three independent experiments, and statistical significance was determined using two-way ANOVA. All the error bars on graphs represent standard deviation of the mean. Sholl analysis was performed by thresholding the mRUBY channels and using the Sholl plugin (ImageJ Fiji) to measure the number of dendritic intersections every 10 μm over a total radius of 150 μm from the cell body. The numbers of intersections were measured from three independent neuronal cultures. Total dendritic length was measured using the Simple Neurite Tracer plugin (ImageJ Fiji) to calculate the total dendrite length of a neuron based on filling neurons with mRUBY. Traces were taken from three independent experiments, and statistical significance was determined using one-way ANOVA.

Levels of PICK1 knockdown and molecular replacement were quantified by measuring integrated densities of PICK1 clusters detected using anti-PICK1 in cortical neuronal dendrites using ImageJ (Fiji).

### Co-immunoprecipitations

Cells were lysed in IP buffer (0.5% Triton X-100, 150 mm NaCl, and 20 mm HEPES (pH 7.4)), and 1% of extract was removed for input. The remaining lysate was precleared with protein G-Sepharose beads (GE Healthcare) at 4 °C for 1 h. 400 μg of cell lysates was incubated with 2 μg of anti-PICK1 (NeuroMab clone L20/8), anti-FLAG (Sigma, F3165), or control IgG (Thermo Fisher, 31903) antibodies and pulled down with protein G-Sepharose beads (GE Healthcare) at 4 °C for 1 h. Beads were washed three times (1 min each) in 1 ml of IP buffer at 4 °C. Bound proteins were detected by Western blotting. For co-IPs carried out under defined [Ca^2+^]_free_, 8–10 6-cm dishes of cortical neuronal cultures were pooled together in 1 ml of co-IP buffer with zero Ca^2+^. 1% of this pool was taken as input, and the remaining extract was equally divided and diluted into 5 ml of co-IP buffer with the appropriate [Ca^2+^]_total_ and [EGTA] to give the stated [Ca^2+^]_free_. Therefore, these experiments share the same input. Defined [Ca^2+^]_free_ were determined using the Max chelator software (Stanford).

### GST and His_6_ pulldowns

Recombinant proteins were expressed and purified from BL21 bacterial cultures. 1 liter of bacterial culture was induced for 2 h using 0.2 mm isopropyl 1-thio-β-d-galactopyranoside at 30 °C. The bacteria were then pelleted and resuspended in 50 ml of HTG buffer (50 mm HEPES, 150 mm NaCl, and 10% glycerol). Bacteria were sonicated on ice, and debris was removed by centrifugation. The resulting supernatant was incubated with glutathione-Sepharose 4B beads (GE Healthcare) or nickel-nitrilotriacetic acid beads (Qiagen) for 1 h at 4 °C. The beads were washed with 1 ml of HTG buffer three times (1 min each). 8–10 6-cm dishes of cortical neuronal cultures or four T75 flasks of transfected HEK293 cells were pooled together in 1 ml of co-IP buffer with zero Ca^2+^. 1% of this pool was taken as input, and the remaining extract was equally divided and diluted into 5 ml of co-IP buffer with the appropriate [Ca^2+^]_total_ and [EGTA] to give the stated [Ca^2+^]_free_. This extract was then incubated with recombinant protein immobilized on beads for 1 h at 4 °C. This was followed by washing the beads three times (1 min each) in 1 ml of the appropriate [Ca^2+^]_free_ co-IP buffers. Bound proteins were detected by Western blotting.

### GFP-trap

For GFP-trap (Chromotek) pulldowns, six wells of a 6-well plate of DIV10 cortical neurons were transfected with pXLG3-GFP PICK1 constructs and harvested 5 days post-transfection. GFP-trap was performed according to the instructions of the manufacturer. Briefly, cells were lysed in 500 μl of lysis buffer (10 mm Tris (pH 7.5), 150 mm NaCl, 0.5 mm EDTA, and 0.5% Nonidet P-40) on ice. 1% was taken as input, and the remaining extract was incubated with 50 μl of GFP-trap beads for 1 h at 4 °C. The beads were then washed with 500 μl of wash buffer (10 mm Tris (pH 7.5), 150 mm NaCl, and 0.5 mm EDTA) three times (1 min each) at 4 °C, and bound proteins were detected by Western blotting.

### Western blotting

Whole-cell lysates or bound proteins from binding experiments were resolved on a 10% SDS-PAGE gel, transferred to PVDF using a wet transfer apparatus, and blocked in 5% milk solution or 5% BSA made up in PBS-Tween. The membranes were blotted with the appropriate primary and secondary antibodies (see below), and bands were visualized using ECL Western blotting substrates (Thermo Fisher Scientific or GE Healthcare). Where appropriate, the membrane was stripped with Restore Western Blot Stripping Buffer (Thermo Fisher) and reprobed. Membranes were incubated with the following primary antibodies overnight at 4 °C: anti-Ago2 (Cell Signaling Technology, clone C34C6; dilution, 1:1000), anti-GluA2 (Synaptic Systems, 182103; dilution, 1:2000), anti-PICK1 (Abcam, ab3420; dilution, 1:1000), anti-FLAG (Sigma, F7425; dilution, 1:2000), anti-c-Myc (Thermo Fisher, clone 9E10; dilution, 1:10,000), anti-GAPDH (clone 6C5; dilution, 1:20,000), anti-GST (Abcam, ab9085), anti-GFP (NeuroMab, N86/8), anti-GluA1 (Millipore, AB1504), and anti-GluA1 Ser(P)-845 (Millipore, AB5849). Secondary antibodies conjugated to HRP were from GE Healthcare and used at 1:10,000 dilutions for 45 min at room temperature. For densitometry, Western blot films were scanned and analyzed in ImageJ, followed by the appropriate statistical analysis. All error bars on graphs represent standard deviation of the mean. For pulldowns and co-IPs, bound proteins were normalized to their respective inputs. For GFP-trap experiments, bound proteins were normalized to the amount of GFP-PICK1 detected by anti-GFP in the pulldown.

### Luciferase assays

DIV12 cortical cultures were co-transfected with the appropriate luciferase constructs and pXLG3-GFP vectors and prepared for luciferase recordings 48 h post-transfection. The Dual-Luciferase reporter assay system (Promega) was used to perform the assays according to the instructions of the manufacturer. Values were normalized for each of at least three independent experiments, and the appropriate statistical analysis was performed. All error bars on graphs represent standard deviation of the mean.

## Author contributions

D. R. and J. G. H. designed the study and prepared the manuscript. D. R. performed the experiments. M. F. and G. T. P. prepared recombinant proteins. D. R. analyzed the data.

## Supplementary Material

Supplemental Data

## Footnotes

2 The abbreviations used are:

miRNA: microRNA
RISC: RNA-induced silencing complex
LTP: long-term potentiation
LTD: long-term depression
NMDAR: NMDA receptor
AMPAR: AMPA receptor
cLTD: chemically induced long-term depression
DIV: days *in vitro*
IP: immunoprecipitation
ANOVA: analysis of variance.
