# Supplementary material for: The PICK1 Ca2+ sensor modulates N-methyl-d-aspartate (NMDA) receptor-dependent microRNA-mediated translational repression in neurons
Source: J Biol Chem. 2017 Apr 12;292(23):9774–86. doi: 10.1074/jbc.M117.776302 (PMC5465499; doi:10.1074/jbc.M117.776302)
Supplement: Supplemental Data [file supp_292_23_9774__index.html]

PICK1 Ca2+ Sensor Modulates NMDA Receptor-Dependent MicroRNA-Mediated Translational Repression in Neurons — The PICK1 Ca2+ sensor modulates N-methyl-d-aspartate (NMDA) receptor-dependent microRNA-mediated translational repression in neurons — Ago2-PICK1 regulation by Ca2+ — Supplemental Data 

# The PICK1 Ca2+ sensor modulates *N*-methyl-d-aspartate (NMDA) receptor-dependent microRNA-mediated translational repression in neurons

## Supplemental Data

- Supplement Index
- Supplemental Data (.pdf, 2.5 MB)
