# Supplementary material for: The PICK1 Ca2+ sensor modulates N-methyl-d-aspartate (NMDA) receptor-dependent microRNA-mediated translational repression in neurons
Source: J Biol Chem. 2017 Apr 12;292(23):9774–86. doi: 10.1074/jbc.M117.776302 (PMC5465499; doi:10.1074/jbc.M117.776302)
Supplement: Supplemental Data [file 10.1074_M117.776302_jbc.M117.776302-1.pdf]

PICK1 Ca^{2+} Sensor Modulates NMDA Receptor-Dependent MicroRNA-Mediated Translational Repression in Neurons

Dipen Rajgor, Maria Fiuza, Gabrielle T. Parkinson and Jonathan G. Hanley.

Supplemental Data:
Figure S-1
Figure S-2

Figure S-1

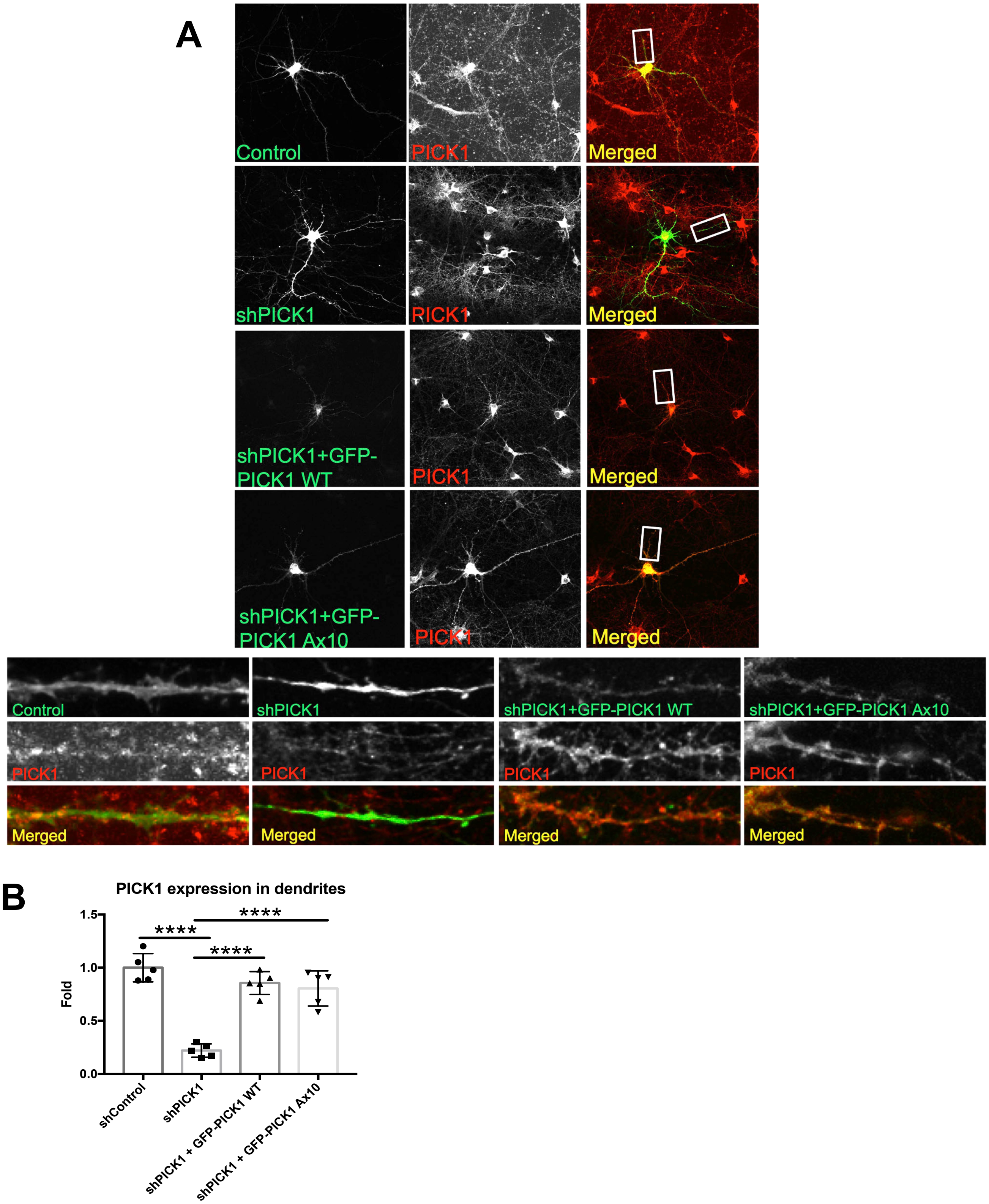

Figure S-1. PICK1 knockdown and expression of molecular replacement constructs in neuronal dendrites

PICK1 levels were analyzed by immunocytochemistry in cortical neuronal dendrites with anti-PICK1 (red) after knockdown by shRNA and expression of shRNA-resistant GFP-PICK1 WT or GFP-PICK1 Ax10.

B) Quantification of PICK1 levels in dendrites. *N=5, ,* **** P<0.0001 one-way ANOVA, Dunnett's post-hoc test.

Figure S-2

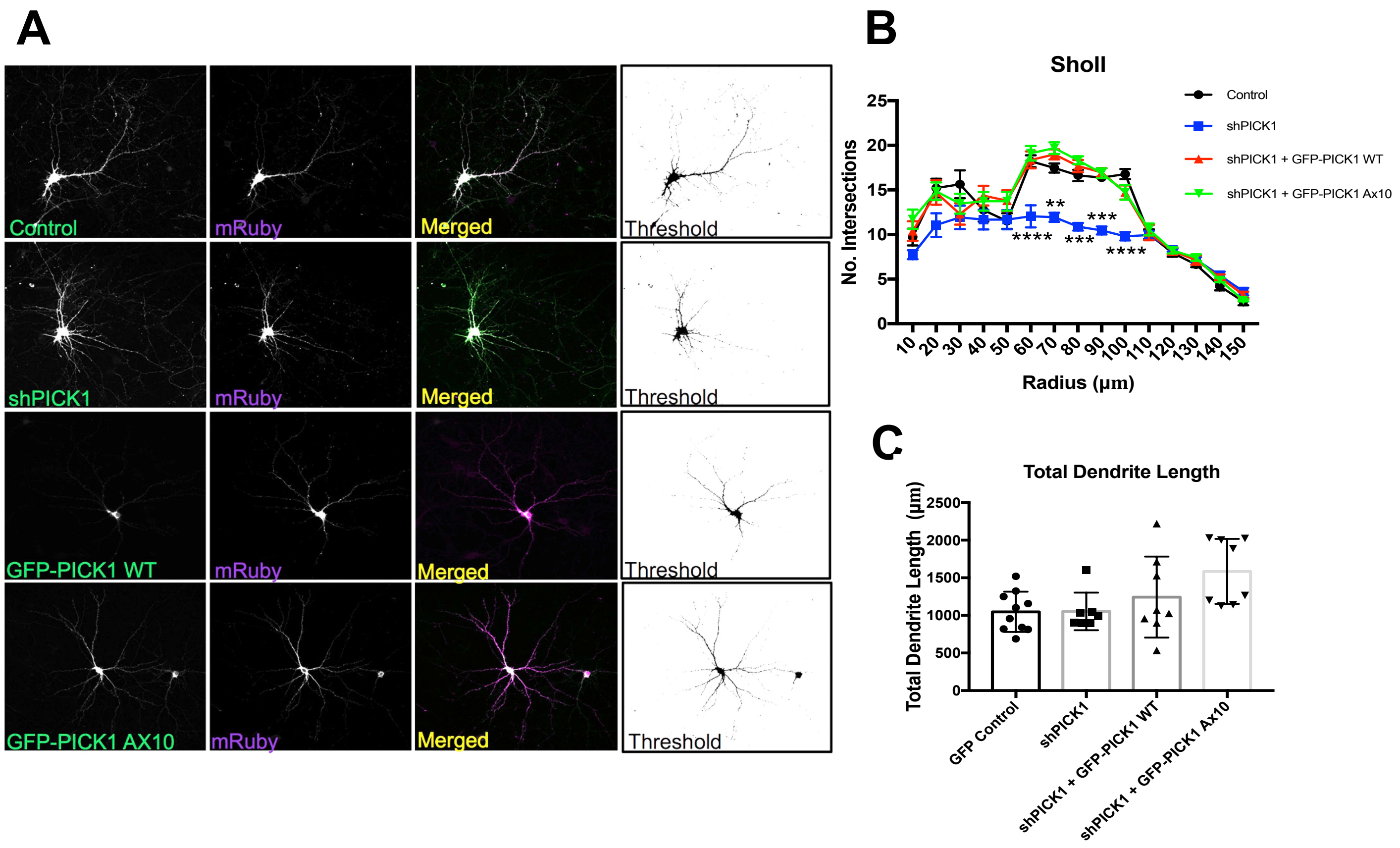

Figure S-2. The effect of PICK1 knockdown and molecular replacement on dendritic branching and length.
A) Representative images of PICK1 molecular replacement constructs co-transfected with mRUBY.
B) Sholl analysis was performed on thresholded images of mRUBY to measure dendritic branching up to 150 µm from cell body. Error bars represent standard error of the mean. *N=15.* ** P < 0.01, *** P < 0.001, **** P < 0.0001 Three-way ANOVA, Tukey's post-hoc test. Statistical significance compared to GFP control.
C) Total dendritic length was measured based on mRUBY filling. *N=7-10,* one-way ANOVA, Dunnett's post-hoc test.
